# Integrating the Metaverse Into Medical Practice: A Systematic Review of Its Potential and Applications

**DOI:** 10.1155/ijta/8397610

**Published:** 2026-08-06

**Authors:** Marsa Gholamzadeh, Shahryar Naji, Elmira Khakvatan, Mohammad Heydari, Saman Mortezaei

**Affiliations:** ^1^ Health Information Management and Medical Informatics Department, School of Allied Medical Sciences, Tehran University of Medical Sciences, Tehran, Iran, tums.ac.ir; ^2^ Students′ Scientific Research Center, Tehran University of Medical Sciences, Tehran, Iran, tums.ac.ir

**Keywords:** augmented reality, digital twin, medical practice, metaverse, mirror worlds, telemedicine, virtual reality

## Abstract

**Introduction:**

The metaverse, characterized by the convergence of virtual and physical realities, represents an emerging concept which offers immersive and interactive experiences with the potential to revolutionize medical care. This study aims to explore the current applications of this technology across various medical specialties.

**Method:**

A systematic search was conducted across five electronic databases including Web of Science, PubMed, Scopus, CINAHL, and IEEE, using a structured query in accordance with PRISMA guidelines. A qualitative analysis was conducted to synthesize findings across different categories such as medical domains, types of metaverse, implementation approaches, target groups, and clinical disciplines.

**Results:**

This review included 46 studies published between 2022 and 2024, with South Korea contributing the majority of publications (*n* = 12, 24%). Applications spanned diverse medical domains, notably medical education (*n* = 7, 15.22%), mental health (*n* = 6, 13.04%), and rehabilitation (*n* = 6, 13.04%). Among the included papers that focused on a single type of metaverse, virtual worlds appeared as the most prominent type (*n* = 23, 50.00%), followed by augmented reality (*n* = 8, 17.39%) and mirror worlds (*n* = 4, 8.70%). The majority of papers concentrated on designing and developing metaverse systems, primarily targeting patients (*n* = 24, 47.06%), medical students (*n* = 12, 23.53%), and healthcare providers (*n* = 8, 15.69%). These systems addressed multiple clinical phases, including treatment/intervention (*n* = 17, 36.95%), education (*n* = 10, 21.74%), and patient follow‐up (*n* = 5, 10.87%).

**Conclusion:**

In conclusion, this review suggests that metaverse applications in healthcare are still in the early stages of development, focusing primarily on system design and feasibility rather than proven clinical outcomes. Although emerging technologies such as artificial intelligence and blockchain are emerging, evidence of clinical effectiveness remains limited. This review provides a descriptive roadmap of current trends and implementation frameworks to support prudent, evidence‐based progress in understudied clinical areas.

## 1. Introduction

The metaverse is a concept that encompasses realities beyond our physical confines, representing a world where our life′s information and experiences manifest through virtual and digital spaces. Its creation has garnered significant attention due to the rapid growth of the digital economy, with the metaverse typically regarded as a shared space that integrates a persistent virtual environment with augmented physical reality [1, 2]. This immersive platform, accessible via augmented or virtual reality (VR), enables users—represented by avatars—to communicate, collaborate, socialize, and interact with digital objects and media. Recently, the metaverse has been attracting interest across various fields and industries, including various medical sciences and clinical practices [3, 4].

In recent years, innovative technologies have transformed various fields of medicine and enhanced research and healthcare delivery while improving the quality of patient care. Among these advancements are the application of artificial intelligence in diverse diagnostic areas, the utilization of telemedicine for continuous patient monitoring and timely interventions, and the integration of wearable devices and sensors for tracking vital signs [5]. Additionally, the emergence of VR and its incorporation into medical care have revolutionized the patient experience, aiming to bridge the gap between theory and practice [6, 7]. As traditional healthcare methods evolve, these technological advancements complement and strengthen clinical skills, enhance patient engagement, and foster interdisciplinary collaboration, ultimately driving the shift towards personalized medicine and facilitating clinical decision‐making [8].

Among the emerging technologies, the metaverse stands out as a domain poised for significant transformation in healthcare following the advancement of VR and AR technology. A 2022 review paper indicates that metaverse technologies, including VR, augmented reality (AR), and mixed reality (MR), demonstrated efficacy in mental health diagnosis and treatment, offering solutions to address both the scarcity of mental health professionals and limited healthcare accessibility [9]. Although various studies have explored the application of the metaverse in diverse medical disciplines such as surgery and medical education [10, 11], the specifics of how this technology can be effectively implemented, the extent of its success, and the areas that have yet to embrace the metaverse remain largely unknown to many professionals in the field. This lack of clarity presents both a challenge and an opportunity for ongoing research and development as the medical community seeks to harness the full potential of the metaverse for improving healthcare delivery.

Alongside these opportunities, several challenges remain, including data privacy, governance frameworks, equitable access, and the readiness of healthcare ecosystems to adopt such technologies [12]. Despite increasing interest in metaverse technology across various sectors, there is currently no comprehensive systematic review examining its applications in various medical specialties and subspecialties. The present systematic review aims to identify the current status, potential benefits, and challenges of metaverse technology in medical sciences and clinical practices. The findings are intended to guide researchers in designing future studies and to assist healthcare leaders in making evidence‐based decisions about implementing metaverse technology in this field.

## 2. Method

In our study, the research question is clearly defined and formulated based on the PCC (Population, Concept, Context) framework to investigate the application of the metaverse (the concept) in various medical specialties and subspecialties (the context). The population in this context refers to clinicians, physicians, or medical specialists.

The inclusion criteria were restricted to studies that explicitly used the term “metaverse” (in the title, abstract, or full text). Because the metaverse is an evolving and often ambiguously defined paradigm—frequently overlapping with concepts like VR, AR, MR, and digital twins—imposing this strict terminological boundary was necessary to ensure conceptual consistency across the reviewed literature.

The articles were retrieved from five databases, namely PubMed, Scopus, Web of Science, CINAHL, and IEEE, according to our search strategy. Our search strategy was formulated based on the keywords extracted from the main research question. The literature search covered studies published from database inception to December 2024. No search filters or limits were applied. The Preferred Reporting Items for Systematic Review and Meta‐Analysis (PRISMA) methodology was employed in this study to conduct a systematic review (PRISMA Checklist is available in the Supporting Information) [13]. The search strategy used in each database, along with the number of results, is shown in Appendix A (Table A‐1) of the Supporting Information.

### 2.1. Study Selection and Extraction Criteria

Articles were included if they met the following criteria: (1) The focus of the study was on applying metaverse technology in medical care or different medical specialties. (2) The population includes all clinicians or healthcare providers with any medical specialty or in general medicine. (3) The study covered all types of medical fields. (4) The term metaverse was used in the study. (5) Published in peer‐reviewed journals and conference proceedings. (6) Limited to those published in English. (7) All types of study designs. (8) Studies that received an acceptable quality score based on the employed checklists.

Articles were excluded if they met the following criteria: (1) The paper was not related to the metaverse technology in medical care or did not use the term in the title, abstract, or full text. (2) Book chapters, letters to editors, short briefs, reports, technical reports, theses, book reviews, review studies, or meta‐analyses. (3) Non‐English papers. (4) Publications for which only the abstract is available.

### 2.2. Study Selection and Screening Process

The study selection and screening process was conducted in three stages according to the PRISMA guidelines: title and abstract review, full‐text reading, and data extraction. EndNote v.20 was utilized to ensure the robustness of the screening and reference management process.

After eliminating duplicates from the initial results, the titles and abstracts were reviewed by three authors for their relevance to the research questions and inclusion criteria. Simultaneously, one reviewer randomly screened the studies and rescreened them if necessary. In the next stage, the full texts of the remaining studies were screened independently and blinded to each other to determine eligible articles based on the criteria defined by the four authors. Articles that appeared ambiguous or unclear were reviewed by the designated reviewer. Through brainstorming sessions, data extraction forms were designed to facilitate the analysis of the reviewed studies. Each author independently filled out the extraction form based on predefined categories, after which the next reviewer assessed and validated the extracted data.

### 2.3. Analysis

Extraction forms were designed to gather useful information from each article, including the author and publication date. Due to the heterogeneity of study designs, a meta‐analysis was not feasible. To synthesize the extracted data, we applied a mixed method of quantitative and qualitative analysis method introduced by Sandelowski et al. [14], which was also used in a previous study by Abtahi et al. to achieve the desired results [15]. This method posits that collecting both qualitative and quantitative findings can reveal underlying knowledge.

A structured coding framework was developed to ensure consistent classification of included studies across multiple dimensions, including clinical domain, patient management phase, implementation type, metaverse type, and technical components (software and hardware). Operational definitions for each category were predefined prior to data extraction. The full codebook is provided as Supporting Information (Appendix B).

Data extraction and coding were performed independently by two reviewers. Disagreements were resolved through discussion, and a third reviewer was consulted when consensus could not be reached.

### 2.4. Quality Appraisal

The quality of the included studies was assessed independently by four reviewers using three validated tools and checklists including the Mixed Methods Appraisal Tool (MMAT, Version 2018), the STARE‐HI (Standards for Reporting of Diagnostic Accuracy Studies—Health Informatics) checklist, and the TRIPOD (Transparent Reporting of a Multivariable Prediction Model for Individual Prognosis or Diagnosis) statement. The MMAT was used to evaluate the overall methodological quality across different study designs. In MMAT, each criterion was scored as “met” (1) or “not met” (0), and an overall score was calculated for each study. The core quality criteria of the MMAT for each study design category are presented in Appendix C (Table C‐1) of the Supporting Information. Studies with a total score of 6 or higher out of 10 were considered to have acceptable quality. The two initial screening questions of the MMAT were used only for eligibility screening and were not included in the quality scoring. The STARE‐HI and TRIPOD checklists were applied to assess the completeness of reporting for health informatics and prediction model studies, respectively. For these checklists, the same threshold of ≥ 50% of the maximum possible score was applied to determine acceptable quality. Disagreements between reviewers were resolved through discussion or by consulting a fifth reviewer.

## 3. Results

**Figure 1:**
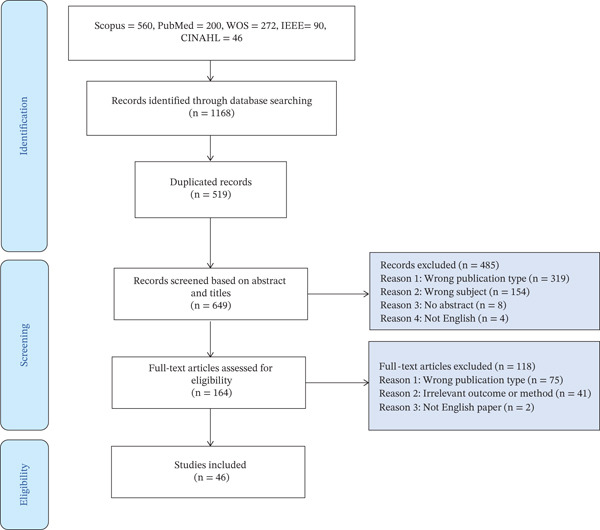
PRISMA flow diagram of the study selection process.

A total of 1168 records were retrieved through the systematic search of electronic databases. Of these, 519 duplicates were removed prior to screening. The remaining 649 records underwent title and abstract screening, which led to the exclusion of 485 records that did not meet the eligibility criteria. From the remaining 164 full‐text articles assessed for eligibility, 118 were excluded for the following reasons: wrong publication type (*n* = 75), the study′s focus not being on metaverse technology in medical care (*n* = 41), and not written in English (*n* = 2). Ultimately, 46 studies met the inclusion criteria and were included in the systematic review. The selection process is illustrated in Figure 1 flow diagram which includes the reasons for excluding articles along with their related numbers.

### 3.1. General Characteristics

**Figure 2:**
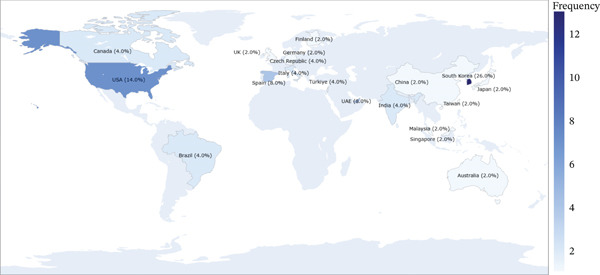
Geographical distribution of the included studies by country.

All the included papers were published after 2022, demonstrating an upward trend in research activity leading up to 2024. The year 2024 accounted for the highest proportion of included publications (*n* = 22, 47.83%), followed by 2023 (*n* = 19, 41.30%), and 2022 (*n* = 5, 10.87%). In terms of geographic distribution, South Korea contributed the largest share of publications (*n* = 12, 24.49%), followed by the United States (*n* = 7, 14.29%); Spain and the United Arab Emirates each accounted for 8.16% (*n* = 4) of the included papers. Notably, two papers (4.08%) were collaboratively authored across multiple countries. A geographical distribution of the included studies is represented in Figure 2. Most of the included papers were journal articles (*n* = 41, 89.13%), whereas only five studies (*n* = 5, 10.87%) were conference papers. The general characteristics of included articles are shown in Table 1.

**Table 1:** General characteristics of included articles.

#	Authors	Year	Country	Clinical aspect	Patient management phase	Implementation type	Metaverse type	Software equipment	Hardware equipment	Results/outcomes
**1**	Cho et al. [16]	2024	South Korea	Nursing	Educating students	Designing and evaluating a metaverse system	Virtual Worlds	ZEPETO platform	Smartphones, tablets, and personal computers	The main application of the metaverse in this study is to develop and implement a family‐centered handoff education program for nursing students using the ZEPETO platform.
**2**	Lee et al. [17]	2022	South Korea	Gastroenterology	Prevention	Metaverse viewpoint assessment	Virtual Worlds	ZEPETO platform	Not specified	High satisfaction rates (95%), increased awareness of early‐onset CRC, and recognition of the need for screening
**3**	Oliveira et al. [18]	2023	Brazil	Healthcare management	Enhancing workflow or modeling clinical workflow	Designing and evaluating a metaverse system	Virtual Worlds	Unity 3D, Blender, and Spatial.io	Head‐mounted displays (HMDs), smartphones, and notebooks	Proposed and developed a prototype, which was evaluated by HCI, XR experts, and stakeholder
**4**	Bazargani et al. [19]	2024	South Korea	Neurological disorder	Early detection and diagnosis	Designing and evaluating a metaverse system	Virtual Worlds	VR environment software (not specified)	Head mounted display	High accuracy in early detection of AD and positive user experience
**5**	Rodriguez‐Florido et al. [20]	2024	Spain	Medical education	Educating students	Designing and evaluating a metaverse system	Virtual Worlds	Unity3D Mirror Networking for multi‐user features.	VR head‐mounted displays and hand controllers. Wireless network.	Implementing metaverse‐based system leads to positive results in reducing technological barriers. High student engagement and improved efficiency in learning anatomy.
**6**	Sai et al. [21]	2024	India	Healthcare management	Patient follow‐up	Designing and evaluating a metaverse system	Virtual Worlds	Harfang3D, Meta Quest 2 VR, and other 3D simulation tools.	Meta Quest 2, da Vinci Research Kit, Reachy robot, and systems with AMD Ryzen 5 4600H processor, NVIDIA GeForce GTX 1650 GPU	Demonstrated enhanced patient interaction in virtual consultations, improved precision in surgical training, and proactive health management through self‐assessment
**7**	Orr et al. [22]	2023	United States, Israel, and Australia	Rehabilitation or physiotherapy	Treatment and clinical intervention	Designing and evaluating a metaverse system	Virtual Worlds	XRHealth therapeutic software, including applications like Balloon Blast, Luna, Mindset, Rotate, and others.	Pico Neo 2 head‐mounted display (HMD) and hand controllers.	Significant improvement in MOLBPDI (17.8% reduction; $\begin{array}{c} p<0.001 \end{array}$). Significant improvement in NDI (23.3% reduction; $\begin{array}{c} p=0.02 \end{array}$). Positive trends in range of motion, pain levels, response times, and functional tests.
**8**	Bueno and Estrela [23]	2022	Brazil	Dentistry	Diagnosis and treatment	System design and evaluation in the metaverse	Virtual Worlds	e‐Vol DX CBCT software with a specific pulp cavity filter for 3D volumetric reconstruction.	CBCT scans were acquired using a PreXion 3D scanner and processed on a PC workstation with an Intel i7‐7700K processor and NVIDIA GeForce GTX 1070 video card.	The method increased the amount of data for detecting accessory root canals and improved visualization of root canal microstructures.
**9**	İbili et al. [24]	2024	Turkey	Medical education	Educating students	System design and evaluation in the metaverse	Augmented Reality	Adobe Illustrator, 3ds Max, Blender, and Substance	Computers and VR headsets	The experimental group outperformed the control group in academic achievement. Students found the metaverse‐based learning enjoyable and effective for deep learning and comprehension.
**10**	JosephNg et al. [25]	2023	Malaysia	Surgery	Treatment and clinical intervention	System design and evaluation in the metaverse	Augmented Reality	SmartPLS	Head‐mounted displays (HMDs)	Improved surgical success rates, enhanced patient trust, and reduced anxiety
**11**	Kulkarni et al. [26]	2024	United States	General medicine	Early detection and diagnosis	System design and evaluation in the metaverse	Mirror Worlds	Tensorflow and PyWavelets	NVIDIA GeForce RTX 2070 SUPER GPU, AMD Ryzen 536,006‐Core Processor, 16GB memory	High prediction accuracy (up to 98.50%) and reduced training time
**12**	Moztarzadeh et al. [27]	2023	Czech Republic	Oncology	Diagnosis and treatment	System design and evaluation in the metaverse	Mirror Worlds	Not specified	Not specified	Demonstrated the feasibility and reliability of digital twins in monitoring, diagnosing, and predicting breast cancer using ML techniques.
**13**	Moztarzadeh et al. [28]	2023	Czech Republic	Dentistry	Diagnosis and treatment	System design and evaluation in the metaverse	Mirror Worlds	MobileNetV2 algorithm	Google Colab environment with an A100‐SXM4–40 GB Nvidia GPU accelerator.	Proposed a blockchain‐based digital twin method for dental diagnosis using MobileNetV2, improving efficiency and reducing costs without extra sensors.
**14**	Pascucci et al. [29]	2024	Italy	Rehabilitation or physiotherapy	Treatment and clinical intervention	System design and evaluation in the metaverse	Virtual Worlds	Unity 2018 game engine, Blender 3.1	Meta Quest HMD, Meta Quest joystick	High usability ratings, sculptures judged more beautiful, longer task completion time for sculptures, and smoother movements for artistic sculptures in the first repetition
**15**	Yang and Kang [30]	2023	South Korea	Psychology/mental health	Treatment and clinical intervention	System design and evaluation in the metaverse	Virtual Worlds	Zepeto World, Google Docs, and Online Learning Management System	Smartphones and tablet PCs	Significant improvement in knowledge, critical thinking ability, and communication ability in the experimental group compared to the control group.
**16**	Sestino and D′Angelo [31]	2023	Italy	General medicine	Patient follow‐up	System design and evaluation in the metaverse	Virtual Worlds	Not specified	Not specified	Higher human‐like interactions positively influence individuals′ intention to use digital‐based healthcare services via perceived anthropomorphism, especially among those with higher emotional receptivity.
**17**	Vila et al. [32]	2024	Spain	Psychology/mental health	Follow‐up and treatment	System design and evaluation in the metaverse	Virtual Worlds	Second Life platform.	Computer with internet connection.	The avatar‐based intervention was effective in treating FOD, improving sexual satisfaction, sexual function, sexual initiative, sexual self‐esteem, reducing sex guilt, and sexual anxiety.
**18**	Necker et al. [33]	2024	United States, Germany	Surgery	Treatment and clinical intervention	System design and evaluation in the metaverse	Virtual Worlds	Microsoft Mesh.	Microsoft HoloLens 2 headset.	Using the metaverse for reconstructive surgery planning and education enhanced collaboration, improved trainee confidence, and provided a more intuitive understanding of anatomy through shared virtual 3D models.
**19**	Moon et al. [34]	2023	South Korea	Rehabilitation or physiotherapy	Treatment and clinical intervention	System design and evaluation in the metaverse	Virtual Worlds	Metaverse platform	Smartphones or tablets with internet connection	MPT showed significant improvements in gross motor function, cardiopulmonary function, and perceived risk of COVID‐19 transmission compared to CPT
**20**	Kangas et al. [35]	2024	Finland	Surgery	Treatment and clinical intervention	Designing and evaluating a metaverse system	Augmented Reality	Unity 3D software development system	VR system was built using the Meta Quest 2 HMD	Most of the experiment participants approved the prototype virtual reality system and expressed interest in using a similar implementation in real orthographic–surgical operation planning cases.
**21**	Vila et al. A. [36]	2024	Spain	Psychology/mental health	Treatment and clinical intervention	Metaverse viewpoint assessment	Mirror Worlds	Not mentioned	Not specified	The results of the analysis revealed a total of four categories and nine subcategories for developing an interventional tool to manage mental health disorders using viewpoints of users.
**22**	Musamih et al. [37]	2024	United Arab Emirates	Neurological disorder	Treatment and clinical intervention	Metaverse viewpoint assessment	Augmented Reality	Decentralized application (DApp)	VR headsets	Integrating blockchain and NFTs into metaverse improves mental health therapy by enhancing engagement, security, and therapy effectiveness
**23**	Kitapcioglu et al. [38]	2024	Turkey	Medical education	Educating students	Metaverse viewpoint assessment	Augmented Reality	VR‐based serious game used in this study, named “3DMedsim ACLS VR”	Not mentioned	Comparing the VR test scores between the MG (Machine‐Guided Virtual Reality‐Based Training) and EG (Educator‐Guided Training in a Metaverse Environment) groups revealed no statistically significant difference.
**24**	Lee et al. [39]	2023	South Korea	Psychology/mental health	Treatment and clinical intervention	Designing and evaluating a metaverse system	Virtual Worlds	(Roblox) and Zoom	Smartwatch (Fitbit charge 5)	Children with HFASD can potentially be familiarized, through metaverse‐based programs, with real‐life social situations to improve sociality and reduce emotional and behavioral problems.
**25**	Cho et al. [40]	2023	South Korea	General medicine	Treatment and clinical intervention	Metaverse viewpoint assessment	Virtual Worlds	MetaForest software	Built‐in camera on the user′s computer	The results of this study support the use of synchronous metaverse programs to treat college students.
**26**	Cho et al. [41]	2023	South Korea	Rehabilitation or physiotherapy	Treatment and clinical intervention	Metaverse viewpoint assessment	Augmented Reality	Not mentioned	Virtual reality‐assisted (VR) treadmill	Technology treadmill walking could improve cognitive function, concentration, muscular endurance, stroke prevention, proper weight maintenance, and cardiorespiratory function.
**27**	Chang et al. [42]	2023	Taiwan	Pediatric	Treatment and clinical intervention	Designing and evaluating a metaverse system	Augmented Reality	The game e designed by authors	A notebook computer equipped with lenses and speakers, and required specifications that could use MR	This study provides the first MR SDM game that has passed the SUS and can be used as an aid in clinical shared decision making (SDM).
**28**	Al‐Zyoud et al. [43]	2022	Canada	General medicine	Early detection and diagnosis	Developing a framework to create a metaverse system	Mirror Worlds, Life Logging	Not mentioned	Digital camera sensor	XGBoost model provided the lowest testing performance error rate (9.5%) in comparison with the MLP and LSTM models
**29**	Oh et al. [44]	2023	South Korea	Neurological disorder	Treatment and clinical intervention	Developing a framework to create a metaverse system	Augmented Reality	Unreal Engine 4 to produce Metaverse contents	Samsung Odyssey VR HMD based on Unreal Engine 4	After the program implementation, the average MMSE‐KC total score of the MCI group increased. Second, the total amount of time spent on content and the number of hints used in the MCI patient group were lowered between before and after treatment.
**30**	Almarzouqi et al. [45]	2022	United Arab Emirates	Medical education	Educating students	Metaverse viewpoint assessment	Not mentioned	Not mentioned	Not specified	The study finds User Satisfaction as an essential determinant of users′ intention to use the metaverse (UMS)
**31**	Ezawa et al. [46]	2023	United States	Psychology/mental health	Treatment and clinical intervention	Designing and evaluating a metaverse system	Not mentioned	Not mentioned	Not specified	The results suggest that metaverse‐based CBI therapy may be a promising approach for treating depression and anxiety.
**32**	Kim et al. [47]	2023	South Korea	Oncology	Follow‐up and treatment	Designing and evaluating a metaverse system	Virtual Worlds, Mirror Worlds, AR, and Lifelogging	Custom‐built metaverse‐based cancer care platform	Not specified	The study suggests that a multidomain metaverse platform can be a valuable tool for improving cancer care and support.
**33**	Mizuta et al. [48]	2024	Japan	Rehabilitation or physiotherapy	Prevention	Designing and evaluating a metaverse system	Virtual Worlds	A custom‐built metaverse platform for the exercise video distribution.	Not specified	The study found that participants in the metaverse group had higher adherence to the exercise program, greater enjoyment of the exercises, and higher levels of physical activity compared to the control group.
**34**	Ryu et al. [49]	2024	South Korea	Nursing	Educating students	Designing and evaluating a metaverse system	Not mentioned	A custom‐built metaverse‐based online learning system	Not specified	The study found that the metaverse‐based learning system was well‐received by the nursing students, who reported high levels of engagement and satisfaction.
**35**	Orr et al. [50]	2023	United States	Psychology/mental health	Treatment and clinical intervention	Designing and evaluating a metaverse system	Virtual Worlds	A custom‐built metaverse platform for the VR‐based interventions.	The study used VR headsets (e.g., Oculus Rift and HTC Vive) for the metaverse‐based interventions.	The study found that the VR and metaverse‐based interventions were effective in reducing the severity of stress and anxiety symptoms among the participants.
**36**	Veras et al. [51]	2023	Canada	Rehabilitations or physiotherapy	Follow‐up and treatment	Developing a framework to create a metaverse system	Virtual Worlds	Not mentioned	Not mentioned	The paper proposes a framework for ensuring equitable access and implementation of virtual rehabilitation in the metaverse era, addressing challenges such as technological barriers, user experience, and ethical considerations.
**37**	Shardeo et al. [52]	2024	India	Healthcare management	Enhancing workflow or modeling clinical workflow	Developing a framework to create a metaverse system	Virtual Worlds, Augmented Reality, Mirror Worlds, and Life Logging	Not mentioned	Not mentioned	The study identified several key enablers that influence the adoption of metaverse technologies in the healthcare sector, including technological, organizational, and environmental factors.
**38**	Alhamad et al. [53]	2022	United Arab Emirates	Medical education	Educating students	Developing a framework to create a metaverse system	Not mentioned	Not mentioned	Not mentioned	The hybrid SEM‐ML approach provided a more comprehensive understanding of the adoption process, with the decision tree analysis supplementing the SEM findings..
**39**	Tsai and Finkelstein [54]	2023	United States	Sport medicine	Patient follow‐up	Designing and evaluating a metaverse system	Virtual Worlds and Life Logging	The study mentions the use of the Arduino IDE for programming the microcontroller and the Unity game engine for the virtual environment.	The study used a Raspberry Pi 4 microcontroller, an Arduino Uno board, a stepper motor, a force sensor, and a display module to implement the virtual biking system.	The study describes the successful design and implementation of a low‐cost virtual biking system using an IoT interface. The system includes features such as resistance control, speed monitoring, and a virtual environment displayed on a screen.
**40**	Salloum et al. [55]	2024	United Kingdom	Dentistry	Educating students	Designing and evaluating a metaverse system	Not mentioned	A custom‐built metaverse platform and AI‐powered virtual agents.	Not specified	The study found that the metaverse‐based training approach, combined with AI‐powered virtual agents, provided an engaging and immersive learning experience for dental students. The students reported improved understanding of dental procedures, increased confidence, and better preparedness for clinical practice.
**41**	Saddik and Ghaboura, [56]	2024	United Arab Emirates	General medicine	Patient follow‐up	Developing a framework to create a metaverse system	Not mentioned	Not mentioned	Not specified	The authors suggest that the integration of ChatGPT′s natural language processing capabilities with the immersive and interactive environment of the metaverse could improve the quality, accessibility, and user experience of medical consultations.
**42**	Huang et al. [57]	2024	China	Medical education	Educating students	Metaverse viewpoint assessment	Not mentioned	Not mentioned	Not specified	The study found that the metaverse‐based training significantly improved the students′ performance in emergency skills, compared to traditional teaching methods. Students reported higher levels of confidence and satisfaction with the metaverse‐based training approach.
**43**	Rudolphi‐Solero et al. [58]	2024	Spain	Medical education	Educating students	Metaverse viewpoint assessment	Virtual Worlds	Not mentioned	Not specified	The average percentage of correct answers in the individual tests and team tasks was $\begin{array}{c} 74.215.1\pm \end{array}$ and $\begin{array}{c} 71.614.7\pm \end{array}$, respectively, without significant differences between both competitions
**44**	Robinson et al. [59]	2023	United States	Addiction medicine	Treatment and clinical intervention	Metaverse viewpoint assessment	Virtual Worlds	Metaverse application called Innerworld	Oculus Quest VR headset and handheld controllers.	Significant increase in positive affect and not find a significant decrease in negative affect and eight major themes
**45**	Yap et al. [60]	2024	Singapore	Pharmacy	Patient follow‐up	Designing and evaluating a metaverse system	Virtual Worlds	ChatGPT, NightCafe, Canva, HeyGen, and Camtasia	Not specified	The majority had experienced the augmented reality (93.8%), lifelogging (87.5%), mirror world (93.8%), and virtual world (75.0%) characteristics.
**46**	Kim et al. [61]	2024	South Korea	Oncology	Early detection and diagnosis	Designing and evaluating a metaverse system	Virtual Worlds	Not mentioned	Glass of VR	Most participants were satisfied with Metaverse Educational Center

The quality assessment of the included studies showed that, for studies assessable with the MMAT, the mean score was 8.45 out of a maximum possible score of 10, indicating generally good methodological quality. For the remaining studies that could not be evaluated with the MMAT, the assessment based on the TRIPOD and STARE‐HI checklists demonstrated that all of these studies achieved acceptable scores. Consequently, all included studies were deemed to be of sufficient quality and were included in the final analysis. The detailed quality appraisal scores for each study based on the MMAT, STARE‐HI, and TRIPOD checklists are provided in Appendix C (Tables C‐2 to C‐3) of the Supporting Information.

### 3.2. Classification of Metaverse Types

Over the past decades, the Acceleration Studies Foundation (ASF) proposed the four types of metaverse as the Metaverse Roadmap (MVR). We followed this roadmap to classify and report all included studies according to this framework. These proposed classifications include AR, life logging, mirror worlds, and virtual worlds [62, 63].

Below, we provide a brief description of each category. AR enhances the physical environment by embedding digital inputs and virtual elements into the real world [64]. Virtual Worlds are immersive, shared digital environments where users, represented by avatars, interact with objects and each other to collaboratively shape and experience the virtual space [65]. A Mirror World is a digital twin of the physical world that allows real‐time interaction with virtual representations of real‐world environments and objects. This MR technology can be accessed through devices like smartphones, tablets, and VR/AR headsets [66]. Life Logging is a metaverse concept that documents personal experiences from daily life, enabling users to share and engage with their personal digital records [67].

According to this framework, among the included studies that focused on a single type, Virtual Worlds emerged as the most frequently implemented (*n* = 23, 50.00%), followed by AR (*n* = 8, 17.39%) and Mirror Worlds (*n* = 4, 8.70%). Additionally, some studies (*n* = 4, 8.70%) explored multiple metaverse types, suggesting broad applicability of this technology, whereas others (*n* = 7, 15.22%) did not specify the type. Additionally, Life Logging was addressed in four studies (8.70%), and often in conjunction with other types, indicating a significantly lower emphasis in the reviewed literature.

### 3.3. Diverse Medical Fields and Applications of Metaverse Technologies in Healthcare

The diverse range of medical fields covered in the included studies highlights the broad applicability of metaverse technologies in healthcare. The included studies were conducted in various fields of medicine including medical education (*n* = 7, 15.22%), psychology/mental health (*n* = 6, 13.04%), rehabilitation or physiotherapy (*n* = 6, 13.04%), general medicine (*n* = 5, 10.87%), dentistry (*n* = 3, 6.52%), healthcare management (*n* = 3, 6.52%), neurological disorder (*n* = 3, 6.52%), oncology (*n* = 3, 6.52%), surgery (*n* = 3, 6.52%), nursing (*n* = 2, 4.35%), addiction medicine (*n* = 1, 2.17%), gastroenterology (*n* = 1, 2.17%), pediatric (*n* = 1, 2.17%), pharmacy (*n* = 1, 2.17%), and sports medicine (*n* = 1, 2.17%). As is apparent, medical education accounted for the largest proportion of studies.

### 3.4. Implementation Types and Related Clinical Applications of Metaverse‐Based Studies

The studies included in this systematic review were implemented for three types of purposes: design and evaluation (*n* = 30), examining the user perspectives on developed system or metaverse technologies (*n* = 10), and framework development (*n* = 6).

The majority of studies (65.22%) focused on the “design and evaluation of metaverse‐based applications” in medical practice. These studies were conducted to design and evaluate a system that covered various clinical disciplines, including rehabilitation/physiotherapy (*n* = 4, 8.69%) [22, 29, 34, 48], psychology/mental health (*n* = 5, 10.86%), surgery (*n* = 3, 6.52%), medical education (*n* = 2, 4.34%), dentistry (*n* = 3, 6.52%), general medicine (*n* = 2, 4.34%), oncology (*n* = 3, 6.52%), nursing (*n* = 2, 4.34%), healthcare management (*n* = 2, 4.34%), neurology (*n* = 1, 2.17%), sports medicine (*n* = 1, 2.17%), pediatrics (*n* = 1, 2.17%), and pharmacy (*n* = 1, 2.17%). The applications within this category targeted different clinical phases, including treatment and intervention (*n* = 11, 23.91%), education (*n* = 5, 10.86%), patient follow‐up (*n* = 4, 8.69%), diagnosis and treatment (*n* = 3, 6.52%), early detection and diagnosis (*n* = 3, 6.52%), workflow enhancement (*n* = 1, 2.17%), and prevention (*n* = 1, 2.17%).

In a total of 10 studies examining user perspectives, the authors focused on topics such as acceptance, feasibility, and usability of metaverse applications in various medical domains. These studies were conducted in various clinical fields including medical education (*n* = 4, 8.69%), psychology/mental health (*n* = 1, 2.17%), neurological disorders (*n* = 1, 2.17%), gastroenterology (*n* = 1, 2.17%), general medicine (*n* = 1, 2.17%), rehabilitation/physiotherapy (*n* = 1, 2.17%), and addiction medicine (*n* = 1, 2.17%). The clinical phases addressed in this category included education (*n* = 4, 8.69%), treatment and intervention (*n* = 5, 10.86%), and prevention (*n* = 1, 2.17%).

Six studies (13.04%) were devoted to “framework development,” aiming to establish conceptual models, guidelines, and strategies for integrating the metaverse into medical practice. These studies focused on general medicine (*n* = 2, 4.34%), healthcare management (*n* = 1, 2.17%), neurological disorders (*n* = 1, 2.17%), rehabilitation/physiotherapy (*n* = 1, 2.17%), and medical education (*n* = 1, 2.17%). The clinical phases addressed in this category included early detection and diagnosis (*n* = 1, 2.17%), workflow enhancement (*n* = 1, 2.17%), patient follow‐up (*n* = 1, 2.17%), education (*n* = 1, 2.17%), and follow‐up and treatment (*n* = 1, 2.17%).

Overall, “design and evaluation” was the predominant implementation type, highlighting the emphasis on developing and testing metaverse applications in real‐world medical settings. Studies under “user perspective analysis” provided insights into user adoption, whereas those under “framework development” contributed to foundational strategies for future implementations.

### 3.5. A Qualitative Classification of Patient Management Phases in Metaverse‐Based Medical Applications

A qualitative classification was derived based on different phases of patient management after reviewing the articles. This classification reflects key aspects of patient management in medical practice, as employed in the reviewed studies including treatment and clinical intervention (*n* = 17, 36.95%), educating students (*n* = 10, 21.74%), patient follow‐up (*n* = 5, 10.87%), early detection and diagnosis (*n* = 4, 8.70%), diagnosis and treatment (*n* = 3, 6.52%), follow‐up and treatment (*n* = 3, 6.52%), enhancing or modeling clinical workflow (*n* = 2, 4.35%), and prevention (*n* = 2, 4.35%) phases.

**Figure 3:**
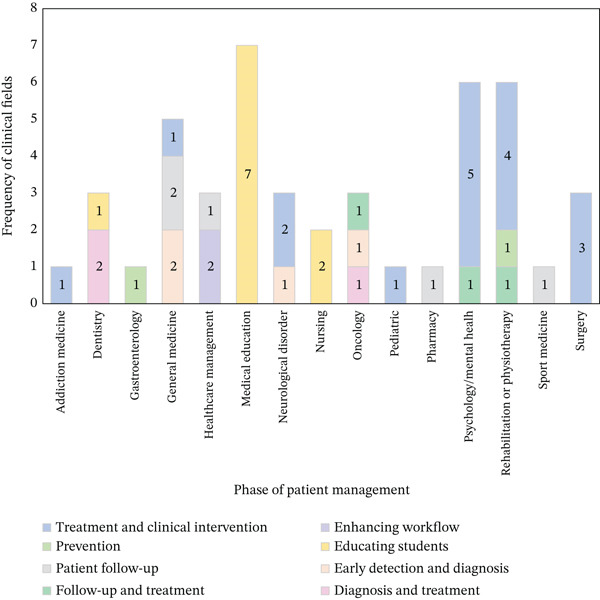
Distribution of metaverse applications across clinical fields and patient management phases.

Diagnosis and early detection ensure timely intervention, whereas treatment and clinical intervention address disease management. Follow‐up and prevention focus on long‐term care and risk reduction. Educating students supports medical training, and workflow enhancement improves healthcare efficiency. Together, these categories provide a structured approach to optimizing patient care. The reviewed articles were investigated in terms of clinical fields and various patient management phases, as represented in Figure 3.

**Figure 4:**
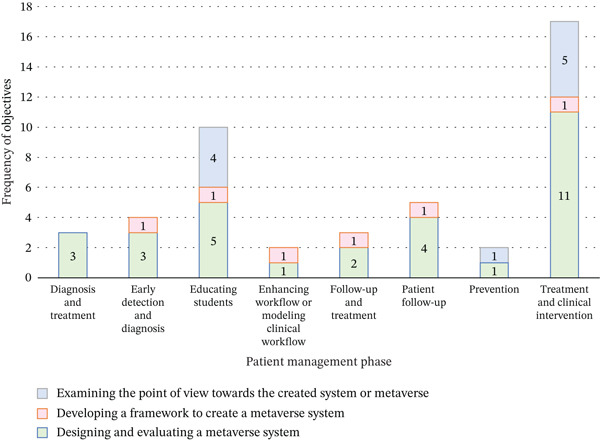
Distribution of implementation types (design/evaluation, user perspective analysis, and framework development) across patient management phases in metaverse‐based healthcare studies.

As we mentioned in the previous section, the included studies were conducted based on three main approaches. The analysis of our findings in terms of these approaches in various patient management phases is shown in Figure 4.

### 3.6. Target User Groups of Metaverse Systems in Healthcare

One key aspect of metaverse systems that needs to be examined is the users they primarily target. Among the included studies, patients were the most frequently targeted group (*n* = 24, 47.06%). Medical students (*n* = 12, 23.53%) and healthcare providers (*n* = 8, 15.69%) constituted the second and third most frequently targeted groups, respectively. Other user groups that were the focus of included studies comprised general population (*n* = 3, 5.88%), populations at risk (*n* = 2, 3.92%), elderly individuals (*n* = 1, 1.96%), and medical educators (*n* = 1, 1.96%). Notably, four studies (8.70%) targeted multiple user groups demonstrating the potential for inclusive solutions across diverse demographics.

### 3.7. Platforms and Software Utilized in Metaverse‐Based Healthcare Systems

To understand the flexibility and diverse approaches implemented in metaverse systems, identifying the various platforms and software utilized in the included studies is crucial. Five studies (10.87%) developed custom‐built metaverse systems, each tailored to address unique healthcare requirements, such as medical training and mental health. Meanwhile, three studies (6.52%) utilized the ZEPETO platform, as their metaverse system specifically targeting pediatric patients, cancer patients, and mental health applications. Additionally, other pre‐existing platforms, including Spatial.io, Second Life, Microsoft Mesh, Roblox, and Innerworld, were each used by one study, demonstrating varied approaches in integrating metaverse technologies into healthcare. The remaining studies did not specify the metaverse platform employed (71.74%).

### 3.8. Hardware Utilized in Metaverse‐Based Healthcare Systems

One key aspect of developing metaverse systems is the variety of hardware that can be utilized. The included studies employed various devices to implement the metaverse system. Meta Quest/Oculus devices were utilized in five studies (10.87%). Other devices, such as the Microsoft HoloLens 2, HTC Vive, Pico Neo 2 HMD, and Samsung Odyssey VR HMD, were each employed in one study. Six studies did not specify the type of AR/VR device utilized (13.04%), and the remaining papers did not report the use of any AR/VR equipment (*n* = 31, 67.39%).

## 4. Discussion

As healthcare continues to evolve with technological advancements, understanding the implications of the metaverse—a new and innovative technology—is crucial for medical professionals, patients, and policymakers seeking to transform medical research, healthcare delivery, and patient care across various clinical domains. Hence, this study aimed to explore the potential applications of the metaverse in medical fields through a systematic review. The objective was to identify the applications of the metaverse in medical practice and to address the necessary software and equipment required for its implementation. Our findings indicate a significant global growth in research and development related to the metaverse, particularly in the healthcare domains. Among the leading countries, South Korea has emerged as a pioneer in this field, followed closely by the United States. The prominence of the United States in advancing metaverse applications in medical fields aligns with the findings of studies by Jinlu Shen and Garavand, which corroborate our results. This highlights the increasing adoption and integration of metaverse technologies in healthcare practices worldwide [68, 69].

Regarding the objectives of the conducted studies, most studies on the metaverse in healthcare domains have concentrated on developing and evaluating metaverse‐based systems aimed at enhancing medical practice. This reflects a growing interest in leveraging immersive technologies to address challenges in healthcare delivery, education, and patient care. However, metaverse‐based systems usually combine cutting‐edge technologies like AI for personalized care, AR for interactive learning, and blockchain for secure data sharing. These integrations enable precise diagnostics, immersive training experiences, and efficient healthcare management. Subsequently, examining users′ views on this new technology is another topic that has attracted the attention of researchers [12, 70]. This highlights the growing interest in understanding how users (e.g., healthcare professionals, patients, and policymakers) perceive and interact with metaverse technologies in medical domains. Another aspect of the studies involved framework development aimed at creating conceptual models, guidelines, and strategies for integrating the metaverse into medical practice. This may be because developing conceptual frameworks and guidelines is essential for integrating metaverse technologies into medical practice, as these frameworks provide a structured approach to implementation and ensure consistency across different healthcare settings and address infrastructure needs. In this regard, Wolko also addressed the strategic integration of metaverse technologies into healthcare in their study, specifically emphasizing their capacity to improve operational effectiveness in medical practice [71].

A key finding from the analysis is the widespread use of the metaverse in medical education, providing realistic simulations for surgical training and interactive learning environments. The present findings seem to be consistent with other research which found that the metaverse revolutionizes medical education by enabling global collaborative learning experiences and transforming professional training [72]. Our findings showed that metaverse provides access to diverse medical cases, including rare scenarios, enhancing students′ understanding of various conditions and treatments, a finding that is further supported by Ghaempanah et al. [11], which explores the impact of the metaverse on medical science education and underscores the need to integrate it into all areas of medical sciences as a supplement to existing evidence.

Following closely, psychology and rehabilitation domains benefit from metaverse interventions to improve cognitive and motor functions. Early‐stage studies propose that the metaverse may offer significant benefits in mental health and rehabilitation by providing immersive, interactive environments that could potentially enhance treatment processes. In mental health, the metaverse facilitates the creation of controlled therapeutic spaces, allowing for personalized interventions and increased patient engagement through metaverse technologies [73, 74]. These findings are in agreement with those obtained by Cerasa et al. [75]. In rehabilitation, particularly poststroke exercises, the metaverse enables educational and immersive capabilities to perform rehabilitation exercises effectively. The study by Cho et al. examined the possibilities and benefits of using the metaverse in exercise rehabilitation, which is consistent with the results of our study [41]. Our analysis has shown that although the metaverse has been used in various medical fields, there are still various areas that need more attention.

**Figure 5:**
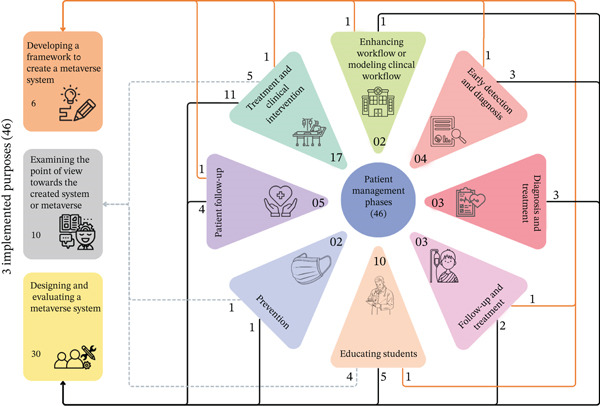
Patient management phases categorized by implementation purpose. The three implementation types (design/evaluation, user perspective analysis, and framework development) are mapped to corresponding clinical phases.

Patient management in clinical practices involves several phases. According to our review, the metaverse is increasingly being used to enhance these phases, particularly in the treatment and clinical interventions phase. Similarly, Mohammed and Albarrak [76] stated that the metaverse is theorized to offer immersive technologies like VR and AR that could improve patient engagement and adherence to treatment plans. In addition, the metaverse facilitates remote monitoring and follow‐up, allowing healthcare providers to track patient progress more effectively. Consistent with the literature, this is particularly beneficial for patients with mobility issues or those living in remote areas, as it increases access to healthcare services. Thirdly, the metaverse can bridge the gap between theoretical education and interventions by providing a worthy educational platform to exercise young doctors [77]. Interestingly, the study also found that the metaverse can enhance and streamline clinical workflows. This aligns with the findings of Ganapathy [78], who similarly highlighted the impact of the metaverse on clinical workflows. In this regard, Figure 5 illustrates the patient management phases categorized by implemented purposes.

As stated in various studies, metaverse in medicine encompasses various components, including virtual worlds, AR, and Mirror Worlds, which are immersive and shared digital environments. In these environments, users, represented by avatars, interact with objects and each other to collectively shape and experience the virtual space. Notably, these components were frequently utilized in studies, as highlighted by Herath et al. and Kilic [79, 80]. Additionally, recent conceptual work has examined how the expansion of virtual care may transform the nature of clinical spaces and professional practice. For example, the concept of the “Mediverse” has been proposed to describe an emerging virtual clinical environment in which the organization of care, professional identity, and patient experience may differ from those of traditional clinical settings [81].

During the development phase of metaverse platforms, the Korean platform ZEPETO emerged as the most commonly used platform across the studies. Other platforms, such as Spatial.io, Second Life, Microsoft Mesh, Roblox, and Innerworld, were also employed. For instance, Hong et al. [82] utilized ZEPETO to develop their product in a study focused on radiology education, whereas Cho et al. [16] and Cho [40] also used the same platform to develop their system in nursing education.

A notable underexplored domain identified in this review is the application of the metaverse in forensic and legal medicine. A recent review by Ferorelli et al. [83] provides a comprehensive overview of potential metaverse applications in this field. The authors highlight the absence of substantial research despite significant potential, particularly in areas such as virtual autopsy training, crime scene reconstruction, forensic case simulation, and digital evidence analysis. In this regard, the recently published FORCE‐XR reporting framework [84] provides a minimum reporting set specifically designed to address auditability, reproducibility, data provenance, integrity, chain‐of‐custody, validation, privacy, security, and governance requirements for extended reality and interactive 3D reconstructions in forensic and legal medicine. Although this framework was published after our search window, it offers a valuable foundation for future studies seeking to implement metaverse technologies in medico‐legal contexts. Given this gap, future studies should explicitly investigate medico‐legal applications, including regulatory frameworks, ethical considerations, and the validity of virtual evidence in both clinical and legal contexts, ideally guided by frameworks such as FORCE‐XR.

However, we succeeded to determine the gap of knowledge in metaverse utilization in various clinical domains. This study encountered some limitations. A key limitation of this review is its strict reliance on the use of the term “metaverse” as an inclusion criterion. While this strategy ensured conceptual clarity and a focused analysis of how the “metaverse” concept is specifically being applied in literature, it inevitably led to the exclusion of some relevant healthcare technologies. Another limitation of this study is the potential for bias in the selection of studies included in the systematic review, as the rapid evolution of metaverse technologies may have led to the exclusion of recent developments or emerging applications that did not appear in our search. Furthermore, this study primarily focused on identifying applications and technologies necessary for implementation and identifying research gaps in this area. However, since many studies did not address critical issues such as data privacy, ethical concerns, and accessibility, which are essential for the practical integration of the metaverse into healthcare systems, it was not possible to comprehensively examine these challenges. Also, another sociotechnical consideration like data privacy, governance frameworks, equitable access, and the readiness of healthcare ecosystems to adopt such technologies need to be addressed in future research. These limitations highlight the need for further research to provide a more comprehensive understanding of the implications of the metaverse in medical settings.

## 5. Conclusion

This integrated review maps the current landscape of metaverse applications across diverse healthcare domains, highlighting a rapid increase in development between 2022 and 2024. Because the included studies predominantly focus on system design, prototyping, and feasibility assessments rather than rigorous clinical trials, the findings of this review should be interpreted as a descriptive overview of development trends rather than definitive proof of clinical effectiveness. While early applications in medical education, mental health, and rehabilitation show promising feasibility and user acceptance, cautious interpretation is warranted regarding their actual impact on patient outcomes. Therefore, while this review provides a valuable foundation for researchers to identify research gaps and infrastructure requirements, definitive claims regarding the widespread and effective integration of metaverse‐based systems into routine clinical practice remain premature. Future research must prioritize high‐quality clinical trials and outcome evaluations to determine the true efficacy of these technologies in medical settings.

## Author Contributions

**Marsa Gholamzadeh:** conceptualization, investigation, writing – original draft, writing – review and editing, supervision. **Shahryar Naji:** investigation, writing – original draft, writing – review and editing, formal analysis. **Elmira Khakvatan:** investigation, writing – original draft, writing – review and editing, formal analysis. **Mohammad Heydari:** investigation, writing – original draft, writing – review and editing, formal analysis. **Saman Mortezaei:** investigation, writing – original draft, writing – review and editing, formal analysis.

## Funding

No funding was received for this manuscript.

## Ethics Statement

The study involves only a review of literature without involving humans and/or animals. The authors have no ethical conflicts to disclose.

## Conflicts of Interest

The authors declare no conflicts of interest.

## Supporting Information

Additional supporting information can be found online in the Supporting Information section.

## Supporting information


**Supporting Information 1** **PRISMA Checklist:** A completed PRISMA 2020 checklist documenting the location in which each recommended reporting item is addressed.

**Supporting Information 2** **Appendix A:** Search strategies and number of results for each database.

**Supporting Information 3** **Appendix B:** The full codebook for qualitative analysis.

**Supporting Information 4** **Appendix C:** Detailed quality appraisal scores of included studies using the MMAT, STARE‐HI, and TRIPOD checklists.

## Data Availability

All data generated or processed during this systematic review are included within the article. The search strategies are provided in the Supporting Information.
