# Supplementary material for: Integrating the Metaverse Into Medical Practice: A Systematic Review of Its Potential and Applications
Source: Int J Telemed Appl. 2026 Aug 6;2026:8397610. doi: 10.1155/ijta/8397610 (PMC13448152; doi:10.1155/ijta/8397610)
Supplement: Supplementary file 2 [file IJTA-2026-8397610-s001.docx]

**Appendix C:**

**Table C-1- Quality appraisal criteria of the MMAT (version 2018) for different study designs**

| **Study type** | **Q1** | **Q2** | **Q3** | **Q4** | **Q5** |
| --- | --- | --- | --- | --- | --- |
| **Qualitative** | 1.1. Is the qualitative approach appropriate to answer the research question? | 1.2. Are the qualitative data collection methods adequate to address the research question? | 1.3. Are the findings adequately derived from the data? | 1.4. Is the interpretation of results sufficiently substantiated by data? | 1.5. Is there coherence between qualitative data sources, collection, analysis and interpretation? |
| **RCT** | 2.1. Is randomization appropriately performed? | 2.2. Are the groups comparable at baseline? | 2.3. Are there complete outcome data? | 2.4. Are outcome assessors blinded to the intervention provided? | 2.5 Did the participants adhere to the assigned intervention? |
| **Non-Randomized** | 3.1. Are the participants representative of the target population? | 3.2. Are measurements appropriate regarding both the outcome and intervention (or exposure)? | 3.3. Are there complete outcome data? | 3.4. Are the confounders accounted for in the design and analysis? | 3.5. During the study period, is the intervention administered (or exposure occurred) as intended? |
| **Quantitative** | 4.1. Is the sampling strategy relevant to address the research question? | 4.2. Is the sample representative of the target population? | 4.3. Are the measurements appropriate? | 4.4. Is the risk of nonresponse bias low? | 4.5. Is the statistical analysis appropriate to answer the research question? |
| **Mixed** | 5.1. Is there an adequate rationale for using a mixed methods design to address the research question? | 5.2. Are the different components of the study effectively integrated to answer the research question? | 5.3. Are the outputs of the integration of qualitative and quantitative components adequately interpreted? | 5.4. Are divergences and inconsistencies between quantitative and qualitative results adequately addressed? | 5.5. Do the different components of the study adhere to the quality criteria of each tradition of the methods involved? |

* This table presents the five core quality criteria (Q1–Q5) of the Mixed Methods Appraisal Tool (MMAT, version 2018) for each study design category. Each criterion was scored as 0 (not met) or 2 (met), yielding a maximum possible score of 10 per study. The two initial screening questions (not shown here) were used only for eligibility screening and were not included in the quality scoring.

**Table C-2- Quality appraisal scores of included studies using the MMAT (version 2018) based on five core criteria (maximum score = 10)**

| No | First author | Year | Study type | _Q1_ | _Q2_ | _Q3_ | _Q4_ | _Q5_ | Total score |
| --- | --- | --- | --- | --- | --- | --- | --- | --- | --- |
| **_1_** | _I. Cho, I. Y. et al. [40]_ | _2024_ | _Non-Randomized_ | _Yes_ | _Yes_ | _Yes_ | _Can't tell_ | _Yes_ | 9 |
| **_2_** | _Lee, T.G. et al. [42]_ | _2022_ | _Quantitative_ | _Yes_ | _No_ | _Yes_ | _Can't tell_ | _Yes_ | 7 |
| **_3_** | _Oliveira, E. et al. [43]_ | _2023_ | _Mixed_ | _Yes_ | _Yes_ | _Yes_ | _Yes_ | _Yes_ | 10 |
| **_4_** | _Bazargani, J.S. et al. [44]_ | _2024_ | _Qualitative_ | _Yes_ | _Yes_ | _Yes_ | _Can't tell_ | _Yes_ | 9 |
| **_5_** | _Rodriguez-Florido, M. Á. et al. [45]_ | _2024_ | _Mixed_ | _Yes_ | _Yes_ | _Yes_ | _Yes_ | _Yes_ | 10 |
| **_6_** | _Orr, E. et al. [23]_ | _2023_ | _Quantitative_ | _Yes_ | _No_ | _Yes_ | _Can't tell_ | _Yes_ | 7 |
| **_7_** | _Bueno, M. R. and Estrela, C. [47]_ | _2022_ | _Mixed_ | _Can't tell_ | _Yes_ | _Can't tell_ | _Can't tell_ | _Can't tell_ | 6 |
| **_8_** | _İbili, E. et al. [48]_ | _2024_ | _Mixed_ | _Yes_ | _Yes_ | _Yes_ | _Can't tell_ | _Yes_ | 9 |
| **_9_** | _JosephNg, P. S. et al. [49]_ | _2023_ | _Mixed_ | _Yes_ | _Can't tell_ | _Yes_ | _Yes_ | _Yes_ | 9 |
| **_10_** | _Pascucci, S. et al. [21]_ | _2024_ | _Quantitative_ | _Yes_ | _No_ | _Yes_ | _Yes_ | _Yes_ | 8 |
| **_11_** | _Yang, S. Y. and Kang, M. K. [53]_ | _2023_ | _Non-Randomized_ | _Yes_ | _Yes_ | _Yes_ | _Can't tell_ | _Yes_ | 9 |
| **_12_** | _Sestino, A. and D'Angelo, A. [54]_ | _2023_ | _RCT_ | _Yes_ | _Yes_ | _Can't tell_ | _Yes_ | _Yes_ | 9 |
| **_13_** | _Vila, A. et al. [55]_ | _2024_ | _RCT_ | _Yes_ | _Yes_ | _Yes_ | _No_ | _Yes_ | 8 |
| **_14_** | _Necker, F. N. et al. [56]_ | _2024_ | _Qualitative_ | _Yes_ | _Yes_ | _Yes_ | _Yes_ | _Can't tell_ | 9 |
| **_15_** | _Moon, I. et al. [22]_ | _2023_ | _RCT_ | _Yes_ | _Yes_ | _Yes_ | _No_ | _Yes_ | 8 |
| **_16_** | _Kangas, J. et al. [57]_ | _2024_ | _Quantitative_ | _Can't tell_ | _Yes_ | _Yes_ | _Can't tell_ | _Yes_ | 8 |
| **_17_** | _Vila, et al. A. [58]_ | _2024_ | _Qualitative_ | _Yes_ | _Yes_ | _Yes_ | _Yes_ | _Yes_ | 10 |
| **_18_** | _Kitapcioglu, D. et al. [60]_ | _2024_ | _RCT_ | _No_ | _Yes_ | _Yes_ | _Can't tell_ | _Yes_ | 7 |
| **_19_** | _Lee, J. H. et al. [61]_ | _2023_ | _RCT_ | _Yes_ | _Yes_ | _Yes_ | _No_ | _Yes_ | 8 |
| **_20_** | _Cho, S. et al. [41]_ | _2023_ | _Quantitative_ | _Yes_ | _Yes_ | _Yes_ | _Can't tell_ | _Yes_ | 9 |
| **_21_** | _Cho, K. W. et al. [33]_ | _2023_ | _Mixed_ | _Yes_ | _Yes_ | _Yes_ | _Can't tell_ | _Yes_ | 9 |
| **_22_** | _Chang, L. et al. [62]_ | _2023_ | _Quantitative_ | _Yes_ | _Yes_ | _Yes_ | _Can't tell_ | _Yes_ | 9 |
| **_23_** | _Al-Zyoud, I. et al. [63]_ | _2022_ | _Quantitative_ | _Can't tell_ | _Yes_ | _Yes_ | _Can't tell_ | _Yes_ | 8 |
| **_24_** | _Oh, G. et al. [64]_ | _2023_ | _Non-Randomized_ | _Yes_ | _Yes_ | _Yes_ | _Can't tell_ | _Yes_ | 9 |
| **_25_** | _Almarzouqi, et al. [65]_ | _2022_ | _Quantitative_ | _Yes_ | _Can't tell_ | _Yes_ | _Can't tell_ | _Yes_ | 8 |
| **_26_** | _Ezawa, I. D. et al. [66]_ | _2023_ | _Quantitative_ | _Yes_ | _Can't tell_ | _Yes_ | _No_ | _Yes_ | 7 |
| **_27_** | _Kim, S. et al. [67]_ | _2023_ | _Quantitative_ | _Yes_ | _Can't tell_ | _No_ | _Yes_ | _Yes_ | 7 |
| **_28_** | _Mizuta, R. et al. [20]_ | _2024_ | _RCT_ | _Yes_ | _Yes_ | _Yes_ | _Yes_ | _Yes_ | 10 |
| **_29_** | _Ryu, H. et al. [68]_ | _2024_ | _Mixed_ | _Yes_ | _Yes_ | _Yes_ | _Can't tell_ | _Yes_ | 9 |
| **_30_** | _Orr, E. et al. [69]_ | _2023_ | _Quantitative_ | _Yes_ | _Can't tell_ | _Yes_ | _No_ | _Yes_ | 7 |
| **_31_** | _Veras, et al. [70]_ | _2023_ | _Qualitative_ | _Yes_ | _Yes_ | _Can't tell_ | _Yes_ |  | 9 |
| **_32_** | _Shardeo, V. et al. [71]_ | _2024_ | _Mixed_ | _Yes_ | _Can't tell_ | _Yes_ | _Can't tell_ | _Yes_ | 8 |
| **_33_** | _Salloum, S. et al. [74]_ | _2024_ | _Quantitative_ | _Yes_ | _Yes_ | _Can't tell_ | _Yes_ | _Yes_ | 9 |
| **_34_** | _Saddik, A. E. and Ghaboura, S. [75]_ | _2024_ | _Qualitative_ | _Yes_ | _Yes_ | _Yes_ | _Can't tell_ | _Yes_ | 9 |
| **_35_** | _Huang, H. L. et al. [76]_ | _2024_ | _Non-Randomized_ | _Yes_ | _Yes_ | _Yes_ | _Can't tell_ | _Yes_ | 9 |
| **_36_** | _Rudolphi-Solero, T. et al. [77]_ | _2024_ | _Mixed_ | _Yes_ | _Yes_ | _Yes_ | _Can't tell_ | _Yes_ | 9 |
| **_37_** | _Robinson, N. et al. [78]_ | _2023_ | _Mixed_ | _Yes_ | _Yes_ | _Yes_ | _No_ | _Can't tell_ | 7 |
| **_38_** | _Kim, S. et al. [80]_ | _2024_ | _Mixed_ | _Yes_ | _Yes_ | _Yes_ | _Yes_ | _Yes_ | 10 |

**Note**: The MMAT (version 2018) includes five core criteria for each study design category. Each criterion was scored as 0 (not met) or 2 (met). Therefore, the maximum possible total score is 10. Studies scoring ≥ 6 out of 10 (≥ 60%) were considered to have acceptable methodological quality.

**Table C-2- Quality appraisal scores of included studies using the STARE-HI checklist**

| **Author(s)** | **Year** | **Score** | **Percentage** | **Acceptable** |
| --- | --- | --- | --- | --- |
| Sai, S. et al. [46] | 2024 | 19 | 53% | Yes |
| Musamih, A. et al. [59] | 2024 | 25 | 71% | Yes |
| Yap, K. Y. et al. [79] | 2024 | 27 | 76% | Yes |
| Alhamad, A. Q. et al. [72] | 2022 | 25 | 71% | Yes |
| Tsai, T. and Finkelstein, J. [73] | 2023 | 28 | 79% | Yes |

**Note**: The STARE-HI checklist consists of 34 items. Each item was scored as 1 (met) or 0 (not met). Therefore, the maximum possible total score is 34. Studies scoring ≥ 50% (i.e., ≥ 17 out of 34) were considered to have acceptable reporting quality.

| **Author** | **Year** | **Score** | **Percentage** | **Acceptable** |
| --- | --- | --- | --- | --- |
| Kulkarni, C. et al. [50] | 2024 | 26 | 56.52% | Yes |
| Moztarzadeh, O. et al. [51] | 2023 | 27 | 58.69% | Yes |
| Moztarzadeh, O. et al. [52] | 2023 | 32 | 69.56% | Yes |

**Table C-3. Quality appraisal scores of included studies using the TRIPOD statement**

**Note: The TRIPOD statement consists of 22 items with a maximum possible total score of 64 (items are weighted/scored based on the original TRIPOD checklist). Studies scoring ≥ 50% (i.e., ≥ 32 out of 64) were considered to have acceptable reporting quality.**
