# Supplementary material for: Integrating the Metaverse Into Medical Practice: A Systematic Review of Its Potential and Applications
Source: Int J Telemed Appl. 2026 Aug 6;2026:8397610. doi: 10.1155/ijta/8397610 (PMC13448152; doi:10.1155/ijta/8397610)
Supplement: Supplementary file 3 [file IJTA-2026-8397610-s002.docx]

**Appendix B:**

**Table B-1-** Clinical and Patient management phase Categorization. Studies were classified according to their clinical domain and the primary stage of care targeted by the metaverse-based intervention.

| Dimension | Category | Operational Definition |
| --- | --- | --- |
| Clinical Aspect | Nursing | Studies that involve nursing practice, nursing education, or patient care delivered primarily by nurses. |
| Clinical Aspect | Gastroenterology | Studies that address diseases, diagnostics, or interventions related to the digestive system. |
| Clinical Aspect | Healthcare Management | Studies that focus on healthcare systems, administration, workflow optimization, or organizational processes. |
| Clinical Aspect | Neurological Disorder | Studies that involve neurological conditions (e.g., stroke, Parkinson’s disease, Alzheimer’s disease), including diagnosis, treatment, or rehabilitation. |
| Clinical Aspect | Medical Education | Studies that involve training of medical students, residents, or healthcare professionals using metaverse technologies. |
| Clinical Aspect | Rehabilitation / Physiotherapy | Studies that focus on physical recovery, motor function improvement, or therapeutic interventions. |
| Clinical Aspect | Dentistry | Studies that involve oral health, dental procedures, or dental education and training. |
| Clinical Aspect | Surgery | Studies that address surgical training, planning, simulation, or intraoperative support. |
| Clinical Aspect | General Medicine | Studies that address general clinical care not restricted to a specific specialty or involving multiple conditions. |
| Clinical Aspect | Oncology | Studies focused on cancer diagnosis, treatment, patient support, or oncology education. |
| Clinical Aspect | Psychology / Mental Health | Studies that address mental health conditions (e.g., anxiety, depression, PTSD) or psychological interventions. |
| Clinical Aspect | Pediatrics | Studies that specifically target child or adolescent populations. |
| Clinical Aspect | Sport Medicine | Studies that involve athletic performance, injury prevention, or sports-related rehabilitation. |
| Clinical Aspect | Addiction Medicine | Studies that address substance use disorders and related interventions. |
| Clinical Aspect | Pharmacy | Studies that involve pharmacological education, drug management, or medication-related interventions. |
| Patient Management Phase | Prevention | Interventions aimed at reducing disease risk, promoting health, or preventing onset of illness in healthy or at-risk populations. |
| Patient Management Phase | Early Detection and Diagnosis | Applications focused on identifying diseases at an early stage, including screening, detection, and diagnostic support. |
| Patient Management Phase | Diagnosis and Treatment | Studies where both diagnostic processes and therapeutic interventions are integrated and equally emphasized. |
| Patient Management Phase | Treatment and Clinical Intervention | Interventions primarily aimed at treating a condition, including clinical procedures, therapy delivery, or rehabilitation interventions. |
| Patient Management Phase | Patient Follow-up | Activities conducted after initial treatment, including monitoring, long-term care, and outcome tracking. |
| Patient Management Phase | Follow-up and Treatment | Studies combining ongoing treatment with longitudinal monitoring, where neither component is clearly dominant. |
| Patient Management Phase | Educating Students | Studies focused on training medical students or healthcare professionals rather than direct patient care. |
| Patient Management Phase | Enhancing Workflow / Modeling Clinical Workflow | Studies aimed at improving healthcare processes, system efficiency, or clinical workflow design rather than direct patient-level interventions. |

**Table B-2.** Technology and Implementation Type Categorization. Studies were classified according to metaverse implementation type, metaverse type, and reported software and hardware components.

| Dimension | Category | Operational Definition |
| --- | --- | --- |
| Implementation Type | Designing and Evaluating a Metaverse System | Studies that develop a functional metaverse-based system or application (e.g., platform, prototype, simulation environment) and include some form of evaluation. |
| Implementation Type | System Design and Evaluation in the Metaverse | Studies that explicitly frame their work within an existing metaverse ecosystem or infrastructure rather than presenting a standalone system. |
| Implementation Type | Metaverse Viewpoint Assessment | Studies that analyze, discuss, or evaluate the metaverse concept in healthcare without implementing a system. |
| Implementation Type | Developing a Framework to Create a Metaverse System | Studies that propose structured models, architectures, or frameworks for building metaverse systems without necessarily implementing a full system. |
| Metaverse Type | Virtual Worlds | Fully immersive, computer-generated 3D environments where users interact via avatars in real time. |
| Metaverse Type | Augmented Reality (AR) | Technologies that overlay digital content onto the real-world environment without fully replacing it. |
| Metaverse Type | Mirror Worlds | Digital representations or simulations of the real world that replicate real environments, systems, or processes, often using real-time data. |
| Metaverse Type | Life Logging | Technologies that capture, store, and utilize personal or behavioral data to represent or enhance user presence in the metaverse. |
| Metaverse Type | Not Mentioned | Studies that do not explicitly define or provide sufficient information to determine the metaverse type. |
| Technical Component | Software Equipment | Any digital platform, engine, application, or computational tool explicitly reported as being used to develop, deliver, simulate, or evaluate the metaverse-based intervention or environment in a study. |
| Technical Component | Hardware equipment | Any physical device used to access, deliver, support, or compute the metaverse-based intervention, including user interface devices, sensing devices, and computational infrastructure. |
