# Supplementary material for: Integrating the Metaverse Into Medical Practice: A Systematic Review of Its Potential and Applications
Source: Int J Telemed Appl. 2026 Aug 6;2026:8397610. doi: 10.1155/ijta/8397610 (PMC13448152; doi:10.1155/ijta/8397610)
Supplement: Supplementary file 4 [file IJTA-2026-8397610-s003.docx]

**Appendix A:**

Table A-1- Search strategies and number of results for each database

| Database | Initial search date | Date Searched | Search strategy | Results |
| --- | --- | --- | --- | --- |
| **PubMed** | 24 May 2024 | 18 December 2024 | ("metaverse"[Title/Abstract]) AND (("medic*"[Title/Abstract]) OR ("Medicine"[Mesh]) OR ("Medical Specialt*"[Title/Abstract]) OR ("clinic*"[Title/Abstract]) OR ("Biomedic*"[Title/Abstract])) | 200 |
| **Web of Science** | 26 May 2024 | 20 December 2024 | TS=("metaverse") AND (TS=("medic*") OR TS=("Medicine") OR TS=("Medical Specialt*") OR TS=("clinic*") OR TS=("Biomedic*")) | 272 |
| **Scopus** | 24 May 2024 | 19 December 2024 | TITLE-ABS-KEY("metaverse") AND (TITLE-ABS-KEY("medic*") OR TITLE-ABS-KEY("Medicine") OR TITLE-ABS-KEY("Medical Specialt*") OR TITLE-ABS-KEY("clinic*") OR TITLE-ABS-KEY("Biomedic*")) | 560 |
| **CINAHL** | 25 May 2024 | 18 December 2024 | ((AB "metaverse") OR (TI "metaverse")) AND ((MH "Medicine+") OR (AB "medic*") OR (TI "medic*") OR (AB "Medical Specialt*") OR (TI "Medical Specialt*") OR (AB "clinic*") OR (TI "clinic*") OR (AB "Biomedic*") OR (TI "Biomedic*")) | 46 |
| **IEEE** | 25 May 2024 | 20 December 2024 | (("Document Title":metaverse) OR ("Abstract":metaverse)) AND (("Document Title":medic*) OR ("Abstract":medic*) OR ("Document Title":Medical Specialt*) OR ("Abstract":Medical Specialt*) OR ("Document Title":clinic*) OR ("Abstract":clinic*) OR ("Document Title":Biomedic*) OR ("Abstract":Biomedic*) OR ("Mesh_Terms":Medicine)) | 90 |
